# Synaptic Strengths Dominate Phasing of Motor Circuit: Intrinsic Conductances of Neuron Types Need Not Vary across Animals

**DOI:** 10.1523/ENEURO.0417-18.2019

**Published:** 2019-07-18

**Authors:** Cengiz Günay, Anca Doloc-Mihu, Damon G. Lamb, Ronald L. Calabrese

**Affiliations:** 1 School of Science and Technology, Georgia Gwinnett College, Lawrenceville, GA 30043; 2 Department Biology, Emory University, Atlanta, GA 30322; 3 Department Psychiatry; Center for OCD, Anxiety and Related Disorders; Cognitive Aging and Memory-Clinical Translational Research Program, University of Florida, Gainesville, FL 32611; 4 Brain Rehabilitation Research Center, Malcom Randall VA Medical Center, Gainesville, FL 32611

**Keywords:** animal to animal variability, hirudo medicinalis, intrinsic membrane properties, motor networks, neuron simulation, synaptic variability

## Abstract

Identified neurons and the networks they compose produce stereotypical, albeit individually unique, activity across members of a species. We propose, for a motor circuit driven by a central pattern generator (CPG), that the uniqueness derives mainly from differences in synaptic strength rather than from differences in intrinsic membrane conductances. We studied a dataset of recordings from six leech (*Hirudo* sp.) heartbeat control networks, containing complete spiking activity patterns from inhibitory premotor interneurons, motor output spike patterns, and synaptic strength patterns to investigate the source of uniqueness. We used a conductance-based multicompartmental motor neuron model to construct a bilateral motor circuit model, and controlled it by playing recorded input spike trains from premotor interneurons to generate output inhibitory synaptic patterns similar to experimental measurements. By generating different synaptic conductance parameter sets of this circuit model, we found that relative premotor synaptic strengths impinging onto motor neurons must be different across individuals to produce animal-specific output burst phasing. Obtaining unique outputs from each individual’s circuit model did not require different intrinsic ionic conductance parameters. Furthermore, changing intrinsic conductances failed to compensate for modified synaptic strength patterns. Thus, the pattern of synaptic strengths of motor neuron inputs is critical for the phasing of this motor circuit and can explain individual differences. When intrinsic conductances were allowed to vary, they exhibited the same conductance correlations across individuals, suggesting a motor neuron “type” required for proper network function. Our results are general and may translate to other systems and neuronal networks that control output phasing.

## Visual Abstract

**Figure:**
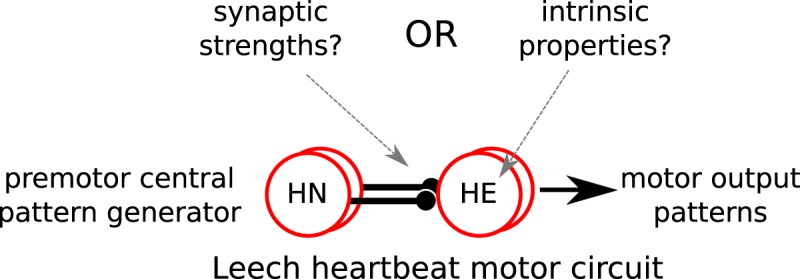

## Significance Statement

Each member of a species is unique, down to its neurons. The ability to experimentally identify a specific neuron across individuals allows investigating the neuron’s variability in spiking activity patterns and therefore its function. Identified neuron types produce stereotypical, albeit individually unique, activity patterns. Uniqueness of activity can be caused by variability observed in the intrinsic properties of neurons and the synaptic connections of the circuit. Here, we propose that, for a rhythmic neuronal motor circuit, the uniqueness derives mainly from differences in synaptic strength rather than from differences in intrinsic membrane conductances. The neuron types that we studied are from the leech heartbeat system. However, our results are general and can translate to other neuronal networks that control output phasing.

## Introduction

Through their projections and synapses onto motor neurons, central pattern generator (CPG) circuits control motor output for a variety of rhythmic behaviors. Across individuals of a species, motor circuits, both motor neurons and CPG elements, show large variability, not only in intrinsic neuronal parameters (Bucher et al., 2005), but also in circuit synaptic parameters (Goaillard et al., 2009). Given this animal-to-animal variability, how do motor circuits produce functional, albeit individually unique, output patterns across animals (Norris et al., 2007a; Goaillard et al., 2009; Williams et al., 2013)?

This question has been largely addressed, not with experimental animals coming from controlled backgrounds, but with those collected from their natural habitats (Goaillard et al., 2009), which have multiple factors that can contribute to biological variability. These genetically unique individuals must adapt to developmental and environmental variability presumably by applying some rules (e.g., homeostatic or developmental) to achieve functional output. We are interested in explaining how such rules act at the neuronal and circuit level to achieve their unique functional network output. In particular, what are the relative contributions of synaptic and intrinsic parameters in the production of unique functional output across animals?

Central pattern generating networks of invertebrates have provided some of the best evidence showing functional output with underlying variability of intrinsic and synaptic conductances (Prinz et al., 2004; Goaillard et al., 2009; Ransdell et al., 2013; Marder et al., 2015; Doloc-Mihu and Calabrese, 2016). In CPGs, the firing phase of component neurons is considered a critical aspect of a functional motor pattern, and phase varies considerably, albeit within functional limits, across animals as shown in the stomatogastric (STG) nervous system (STNS) and in the leech heartbeat system (Wenning et al., 2004a,b; Bucher et al., 2005; Norris et al., 2006, 2007b). For leech heartbeat, each animal arrives at a unique solution to produce a functional heartbeat motor pattern based on phase differences in the premotor pattern and synaptic strength patterns from the CPG premotor interneurons to motor neurons (Norris et al., 2011; Wright and Calabrese, 2011b).

Previous large-scale modeling simulations in other systems, which included both synaptic and intrinsic channel variations, generated databases of different conductance parameter configurations, but these were compared to population averages rather than data collected from individuals (Prinz et al., 2004). Databases of simplified computer models were also constructed to explain variable phasing observed in a multianimal dataset (Williams et al., 2013). In this article, we focus on the coordination of heart motor neurons (HEs) that each receive their inputs from inhibitory premotor heart interneurons (HNs). While all input-output spiking phases and synaptic strengths were recorded, intrinsic conductances in these individuals could not be measured simultaneously. We use computer models to match neuronal profiles (i.e., specific biological instances of network spike phasing and synaptic parameters) to reverse engineer motor neuron intrinsic properties, such as in the case for single animals (Lamb and Calabrese, 2013), but extend this methodology to multiple individuals.

Premotor and motor phasing and synaptic inputs to leech heartbeat motor neurons were experimentally characterized from a set of six individuals (Norris et al., 2007a, b; Wright and Calabrese, 2011b). The bilateral motor circuit was reconstructed by using the same parameterized conductance-based multicompartmental motor neuron model as in Lamb and Calabrese (2013). We optimized sets of model instances (with varying parameter values) of the model neuronal circuit using a multiobjective evolutionary algorithm (MOEA) within the observed natural variability to produce each individual leech’s unique motor output and assessed the relative contribution of intrinsic ionic conductances versus synaptic parameters. We started by keeping synaptic strengths at the measured average values for the individual and allowing only variation of the intrinsic ionic conductances and bilateral electrical coupling between motor neuron pairs. We found that functional models were difficult to find and that they were not successful when tested with the premotor inputs and expected motor output from other individuals in our set. However, allowing also the synaptic strength parameters for an individual to vary within the error of our measurements for that individual led to much higher success rate for our motor neuron model searches. Moreover, such motor neuron models were highly successful when challenged with premotor parameters (synaptic strengths allowed to vary within the measurements’ error) from other individuals. Thus, synaptic strength parameters appear more critical than intrinsic membrane parameters in the expression of animal-to-animal variation in motor output in the leech heartbeat system.

## Materials and Methods

### Electrophysiology and spike-triggered averaging (STA) of synaptic currents

Electrophysiological recordings were obtained from previously published data (Norris et al., 2011). Recordings were made in isolated nerve cords of *Hirudo* sp. extracellularly from premotor HN interneurons and in voltage-clamp from ipsilateral segmental HE motor neurons. Norris et al. (2011) described 12 preparations that have complete recordings of input spiking pattern (premotor interneurons), output spiking pattern (motor neurons) and synaptic strengths in two motor neurons of all four premotor interneurons in both peristaltic and synchronous coordinations (see beginning of Results section for their definitions). They showed statistically that the synaptic strengths of these preparations were representative of a sample involving many more preparations of less complete data from previous studies (Norris et al., 2006, 2007a,b). Of these 12, six were perfect in the sense that there were no changes in period between the recordings of the two coordinations so that data from one side could be reflected to give a bilateral pattern; this was very important for making a bilateral pattern for the modeling done here. The pattern of interneuron activity and motor neuron activity of these six is representative of the 12 and enabled very detailed modeling and dynamic clamp studies that ultimately led to this study (Wright and Calabrese, 2011a,b; Lamb and Calabrese 2013). A recently published meta-analysis of all the preparations (>100) is available in a project database with all data available on Dryad (https://datadryad.org/resource/doi:10.5061/dryad.c0g0p/3; Wenning et al., 2018) enabling external validation of the representativeness of this sample. We show here that the premotor patterns and motor patterns in both coordinations are representative of the much larger sample in the project database (Extended Data Fig. 2-1).

**Figure 1.**
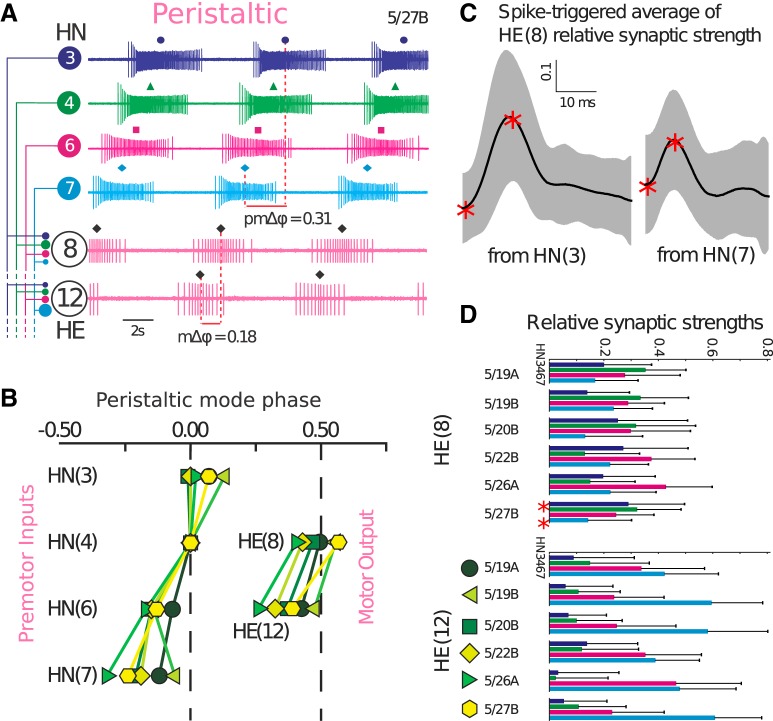
Experimental data from multiple animals show large variability in interneuron phase, output phase, and synaptic strengths. ***A***, Representative ipsilateral connectivity schematic and extracellular traces from preparation 5/27B in peristaltic coordination showing premotor heart interneuron (HN) neurons in segments 3, 4, 6, and 7, with projections to heart motor neurons (HEs) in segments 8 and 12. Phase difference in the premotor inhibitory synaptic input (pmΔ*φ*) from heart interneuron HN(*x*) to HE(*y*) results in a motor phase progression (mΔ*φ_x,y_*). ***B***, Phases of both HNs and HEs vary across preparations. ***C***, STAs of relative synaptic strengths of HN(3) and HN(7) to HE(8) synapses in preparation 5/27B showing the mean (black lines) and SD (gray areas). Red stars show the points between which amplitudes are measured. These two synaptic currents correspond to the red stars in panel ***D***. ***D***, HN to HE relative synaptic strengths (mean ± SD) from six individual animals show variability in their synaptic strengths and disparity between inputs and their ordering of magnitudes.

**Figure 2.**
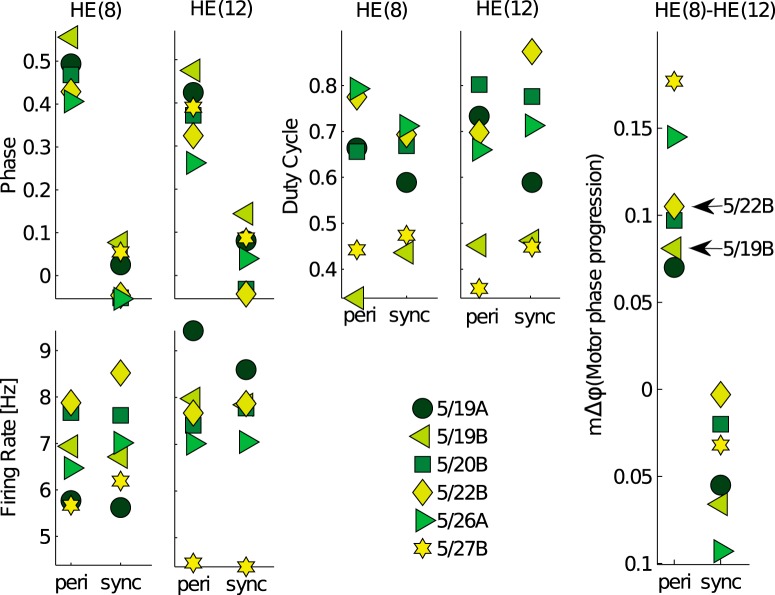
Metric targets for peristaltic (peri) and synchronous (sync) coordinations also varied across six individual leeches. Animal target (5/19B) used for tuning original model (Lamb and Calabrese, 2013) had a relatively small motor phase progression compared to another target (5/22B) of interest (right). These six animals constitute a representative sample from a larger project database. Comparisons of metric distribution between our sample and the project database is shown in Extended Data Figure 2-1.

10.1523/ENEURO.0417-18.2019.f2-1 Extended Data Figure 2-1 Distribution of premotor phase progressions measured as Δϕ HN(4) – HN(7) and motor phase progressions measured as Δϕ HE(8) – HE(12) in our project database (top) and in the six animals in our sample used (bottom) in the current analysis. Top, Distribution of premotor/motor phase progressions across all preparations in the project database. Each symbol on the circular phase plots represents the average intersegmental Δϕ of one preparation in one switch cycle. The number of preparations and the mean intersegmental Δϕ (±SD) is indicated for the CPG pattern (*N* = 129; circles) and of the motor pattern (*N* = 83, triangles) for peristaltic (magenta) and synchronous (blue) coordination, yellow corresponding symbols show the animals in the sample used. Thick arrows represent the average intersegmental Δϕ across preparations (values indicated for each level) and ± their angular SD. The length of the phase vector is also indicated and measures coherence. Bottom, Distribution of premotor/motor phase progressions across the animals in the sample used. Symbols, numbers, vectors as in top. It is important to emphasize that the six animals used in the sample were exceptional recordings in the sense that we were able to measure all phase and synaptic strength parameters considered here, and there was no period change during the recordings. Figure modified and expanded with permission from Wenning et al. (2018). Angela Wenning kindly made the figure. All data is available on Dryad (https://datadryad.org/resource/doi:10.5061/dryad.c0g0p/3). Download Figure 2-1, EPS file.

Recorded electrophysiological activity traces were analyzed using custom scripts written in the MATLAB computing environment (MathWorks). These analysis scripts extracted bursting characteristics of recorded neurons (https://github.com/RonCalabreseLab/Burst-Phase-Matlab-lib). They were also applied to quantify characteristics of simulated neurons (see below). These characteristics were used as the target metrics to evaluate whether model neurons are similar to recorded ones. The analysis started by finding beginning and ending spikes in each burst, their median spikes (indicated with diamonds in figures) that is used to compare phasing of bursts, within-burst spike rate and amplitude. It also low-pass filtered the bursting activity to extract the slow-wave envelope on which the bursts rode. The height of this slow-wave envelope was also one of the target metrics.

For assessing the strength and dynamics of synapses, we have employed an offline spike-triggered averaging (STA) method to analyze IPSCs caused by premotor HNs that are recorded in voltage clamp from HEs, similar to a previous method (Norris et al., 2007a). STA was used to determine synaptic connectivity, strength, and dynamics (short term synaptic plasticity) in both coordination modes, but using data recorded from one side of each preparation. Data from the two coordination modes were combined to increase the signal-to-noise ratio in STA because no significant differences were noted between coordination modes in synaptic connectivity, strength, or dynamics (Norris et al., 2007a).

Spike and IPSC detection/averaging were performed off-line using custom MATLAB software (https://github.com/RonCalabreseLab/HN-HE-synapses-STA). For most of the duration of their bursts, the premotor interneurons tend to fire at a quasi-constant rate, often resulting in multiple peaks in the STA. The largest of these peaks was selected in each trace for measurement of amplitude and latency as before (Norris et al., 2007a). Data used in the STA estimation can be accessed publicly (https://doi.org/10.6084/m9.figshare.7963757).

We have improvements over the previously reported STA method in several aspects: (1) we verified that proper number of leading and trailing IPSCs are removed from the STA in each burst; (2) we have an interactive feature to exclude from averaging IPSCs that are near post-synaptic spikes, which escape from voltage clamp because of poor space clamp of the spike initiation zone; (3) we provide analysis of within-animal variability of the STA and its comparison to the average (Fig. 1*C*); (4) finally, we use an improved method to align the current offset between traces from different bursts. Previous methods reported align traces to have zero current offset at their value that corresponded to the presynaptic spike trigger time of the selected HN. However, the trace could shift up or down considerably with this method based on the coincidental existence of IPSCs from other HNs during this time. Instead of this method, we used a bandpass filter (5–3000 Hz) to remove low-frequency current offsets from traces independent of their value at the spike trigger time. This method reduced the offset visibly and quantitatively allowing more accurate averaging.

### MOEA optimization of a conductance-based ensemble model of HE motor neurons

To simulate HE motor neurons, we used a previously-published multicompartmental conductance-based model (Lamb and Calabrese, 2013; model files available on the ModelDB repository, https://senselab.med.yale.edu/ModelDB/ShowModel.cshtml?model=153355). Each motor neuron in this model is composed of seven compartments: a soma, three serially-connected primary neurite compartments, a secondary neurite compartment (passive only) branching from the primary neurite, a synaptic compartment connected to the secondary neurite, and an axon serially-connected to the end of the primary neurite. Each of these included combinations of intrinsic ionic channels specified in Table 1. The ensemble model of the heartbeat motor neuron circuit contains bilateral pairs of these motor neuron models coupled together electrically with a conductance (*g*_synE_) at their respective synaptic compartments. Two pairs of model neurons are simulated for leech segments 8 and 12. Different segmental HE motor neurons receive their corresponding recorded HN inputs. We modified this model (https://github.com/RonCalabreseLab/HE-model) to allow varying passive parameters independently across different compartments, added variable global and individual synaptic multipliers, and allowed user specification of different HN interneuron input patterns recorded from different leeches in our data set (https://doi.org/10.6084/m9.figshare.7963733). We focused our investigations on one model instance from Lamb and Calabrese (2013), whose maximal conductance parameters are shown in Table 2.

**Table 1.** Intrinsic ion channels and electrical coupling components in model and their maximal conductance parameter’s ceiling value

Name	Channel	*g* (*S/m* ^2^)
Na	Fast sodium	3500
P	Persistent sodium	9.5
CaS	Slow calcium	0.5
K_1_/K_2_	Delayed-rectifier potassium	25 (soma), 375 (neurites), 500 (axon)
K_A_	A-type potassium	50 (neurites), 750 (axon)
K_Ca_	Calcium-dependent potassium	50
synE	Synaptic coupling	10 [nS]

MOEA algorithm optimized parameters between 2% and 100% of these values at 2% increments.

**Table 2.** Maximal conductance parameter as percentages of ceiling values for selected model #47 from set C of Lamb and Calabrese (2013**)**

Conductance	%
Soma K_1_	8
Soma K_2_	92
Neurite K_1_	56
Neurite K_2_	4
Neurite K_A_	32
Neurite P	18
Neurite CaS	72
Neurite K_Ca_	4
synE	22
Axon Na	76
Axon K_1_	4
Axon K_2_	96
Axon K_A_	80

Lamb and Calabrese (2013) had previously used a MOEA approach to optimizing model circuit parameters to find motor patterns that match recordings from a single animal (5/19B). Model outputs were quantified based on measurements of activity waveforms (Table 3). Target ranges from each animal’s target values indicate acceptable metric values during ensemble model fits. We used the same target ranges in this study to maintain consistency and produce comparable results. We have extended this schema to fit model outputs to targets from multiple animals. Target ranges selected can be compared in the table to SDs in peristaltic (peri) and synchronous (sync) coordination mode measurements from HE(8) and HE(12) motor neurons averaged over data recorded from six leeches. Duty cycle and phase metric target ranges were less than one SD over the data set. Frequency had an especially relaxed target range. We ignored the absolute phase metrics and instead used the motor phase progression metric. Motor phase progression depends on the absolute phases of motor neurons because it is the phase difference of HE(8) and HE(12). In total, there were 18 metrics that the optimization algorithm maintained within the target ranges. The analysis was performed using custom MATLAB scripts (https://github.com/RonCalabreseLab/HE-model-analysis-matlab).

**Table 3.** Target ranges (leftmost column) used for optimizing all model circuit metrics (Lamb and Calabrese, 2013**)**

	Target	HE(8)		HE(12)	
Metric	range	Peri, mean ± SD	Sync, mean ± SD	Peri, mean ± SD	Sync, mean ± SD
Duty cycle	0.10	0.61 ± 0.18	0.60 ± 0.12	0.62 ± 0.17	0.64 ± 0.17
Spike frequency (Hz)	7	6.73 ± 0.93	6.94 ± 1.03	7.33 ± 1.63	7.25 ± 1.49
Phase (burst median)	0.03	0.49 ± 0.07	0.00 ± 0.06	0.37 ± 0.08	0.05 ± 0.07
Slow wave height (mV)	5	10	10	10	10
Spike height (mV)	7.50	15	15	15	15
Motor phase progression	0.06	0.11 ± 0.04	−0.04 ± 0.03		

These ranges provided the limits around each animal’s target values for fitting ensemble models. Phase and duty cycle are displayed in normalized units. Metrics of slow wave height and spike height were used to ensure models were physiologic and were estimated from recordings. Target ranges are compared to mean and SD of the measurements across six animals for HE(8) and HE(12) in the columns to the right for peristaltic (peri) and synchronous (sync) coordination modes. Motor phase progression is calculated using phase of both motor neurons, so it was displayed only once under HE(8).

Multiobjective optimization algorithms have been successfully used for conductance model parameter optimization before (Druckmann et al., 2007). The basic idea is to find solutions that optimize each objective separately, instead of previous methods that weigh together different objectives to optimize a summed metric. Optimizing the summed metric may give good solutions in one metric while doing worse in others. The user can specify importance of some metrics by weighing them stronger if known in advance. However, this may skew results in favor of a few metrics. Multiobjective methods behave much more fairly, where solutions are evaluated for their goodness by checking all objectives. Therefore, they are more appropriate for matching biological features, which are all important to match. These methods have become the standard in ambitious neuronal modeling projects (Markram et al., 2015), by accumulating many new advancements (for review, see Druckmann, 2013). One of the most commonly employed MOEA algorithms is the non-dominated sorting genetic algorithm (NSGA-II; Deb et al., 2002). However, this algorithm is known to be unsuitable for problems with more than three objectives (Zou et al., 2008), which makes it a suboptimal choice for our optimization task with 18 output metric objectives. Lamb and Calabrese (2013) have employed a custom modification of NSGA-II that partially eliminated this handicap, the elitist non-dominating vector evaluated genetic algorithm (endVEGA), which was written in the C++ programming language and used a roulette wheel to choose more of the lower-value objective metrics irrespective of their number (Milanova et al., 2006; Smolinski et al., 2007). Since an easily customizable implementation of this algorithm was not publicly available, we adopted a custom open-source MATLAB toolbox called GODLIKE (http://www.mathworks.com/matlabcentral/fileexchange/24838-godlike-a-robust-single—multi-objective-optimizer). We have customized and improved GODLIKE by allowing parallel processing on multithreaded hardware. GODLIKE offered four multiobjective optimization algorithms that can be combined together, and included an NSGA-II implementation. As expected, it failed to converge in our 18-dimensional objective function space, because it could always find solutions where some metric was better, which could not be dominated by any other population member. For example, it could find a model instance that has a perfect match to the target spiking frequency, while several other metrics remained outside of target range. We stopped this overzealous single-objective fitting by preventing the optimization of metrics below a threshold, in particular the limits of our target range for each metric. A fuzzy truncation function on the squared metric value (Extended Data Fig. 3-1*A*) allowed us to place a lower bound on metric errors equal to the target range value, such that they cannot be improved further. Therefore, the algorithm focuses on improving the remaining metrics (Extended Data Fig. 3-1*B*). In practice, this method did not dramatically improve the quality of solutions found, but it did improve the speed of convergence and the number of evaluations to reach good quality (low target metric error) solutions (Extended Data Fig. 3-1*C*,*D*). That is, many more low maximal absolute error (MAE) solutions were found with same number of evaluations. Control of simulations, comparison and sorting of metrics, and plotting were achieved using the Pandora toolbox (Günay et al., 2009; RRID: SCR_001831) for MATLAB. Figures in this article can be generated using the public resources for the source code (https://github.com/RonCalabreseLab/HE-model-animals-figures) and the associated simulation data (https://doi.org/10.6084/m9.figshare.7963748).

**Figure 3.**
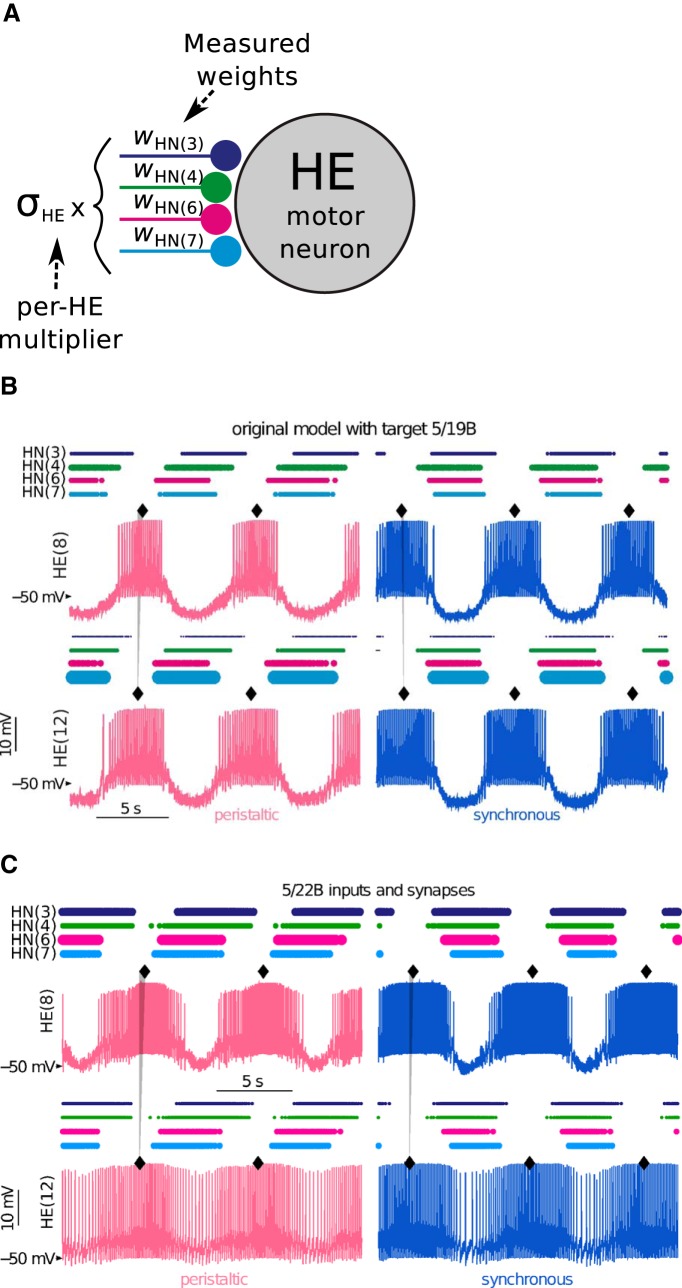
Multicompartmental conductance model of heart motor neuron circuit simulated with synaptic multipliers. ***A***, A global multiplier (*σ*) is required for scaling relative synaptic current measurements from individual HE motor neurons. ***B***, Lamb and Calabrese (2013) evolved a population of 431 model instances with different intrinsic conductance parameters sets that successfully produced a motor phase progression for animal 5/19B (with target mΔ*φ* = 0.081) within target range of 0.06 (for details of method, see Extended Data Fig. 3-1). Sample model instance (#47) shows bursting patterns where diamonds mark the middle spike of the bursts and the gray triangle connecting the traces of HE(8) to HE(12) indicate the motor phase progression. Peristaltic and synchronous coordination modes are indicated with magenta and blue traces throughout this article. The horizontal sequence of dots atop traces aligned with HN interneuron labels indicate their firing times, while the dots’ thicknesses indicate the relative strength between the HN input onto the corresponding HE motor neuron. ***C***, When *σ*-scaled (*σ*_HE8_ = 0.73 and *σ*_HE12_ = 0.75) HN firing patterns of a different animal (5/22B, with target mΔ*φ* = 0.105 ± 0.06) were simulated as input in the HE circuit model, it resulted in a smaller mΔ*φ* = 0.008, which failed to reproduce the increased target value. Extended Data Figure 3-2 gives more details about the impact of the *σ* factors on the activity characteristics. Furthermore, searching across all maximal conductances (of intrinsic ionic currents and bilateral electrical coupling between HEs) and HE synaptic scaling parameters of the model failed to find any solutions within one target range for all metrics for preparation 5/22B (Extended Data Fig. 3-3).

10.1523/ENEURO.0417-18.2019.f3-1 Extended Data Figure 3-1 MOEA improvements. ***A***, A fuzzy filter was used to eliminate number of solutions that fall within one target range of the metrics. Any improvement of metric difference lower than the unit range is not rewarded because the fitness value has a minimum of 1. ***B***, The cartoon demonstrates the two-dimensional case, where fitness values that fall within the unit box are assumed “good enough.” They no longer need to be improved or considered new points on the Pareto front [e.g., all points inside the box will map to (1,1) and will be indistinguishable]. This allows the algorithm to focus on minimizing the other metrics (i.e., in the direction of the arrow). ***C***, ***D***, Example parameter searches across the two-dimensional space of HE(8) and HE(12) global synaptic multipliers (σ). Comparison of 189 evaluations of search by GODLIKE ASA+GA algorithms (***C***) versus the same search method repeated with the fuzzy fitness metric in panel ***A*** for 307 evaluations (***D***). The color bar shows the MAE in all metrics (i.e., worst error). Only dark blue colors indicate the desired solution region where all metrics are within one desired range of the target. In the left panel (***C***), very few model instances were in the unit target range. However, in right panel (***D***), most of the model instances focused in the desired unit target range. Therefore, truncating the objective space complexity using the fuzzy method improved the ratio of converged solutions into unit region and therefore the speed of convergence. Download Figure 3-1, EPS file.

### Statistics

Synaptic strengths measured from living animals and computer simulations were compared using the Kruskal–Wallis test. Being a non-parametric test, Kruskal–Wallis does not assume samples follow a specific distribution. It tests the null hypothesis that medians between the two distributions are equivalent. We chose this test as it is more stringent than a one-way ANOVA test, which assumes the underlying distributions are normal. The distributions of the living and simulated synaptic values were shown in detail in a supplementary figure (Extended Data Fig. 6-2). The Kruskal–Wallis test was applied using the implementation found in MATLAB (MathWorks) named “kruskalwallis.”

Correlations between intrinsic P and K_2_ conductances were tested using multiple linear regression in MATLAB (MathWorks) with the “regress” command. *R* ^2^ and *p* statistics were reported from the output of this command.

## Results

### Experimental data from multiple animals vary in interneuron phase, output phase, and synaptic strengths

The leech heartbeat is a bilateral system, where blood is pumped by rear-to-front peristalsis on one side (heart tube) and nearly synchronously on the other side (heart tube). This activity pattern of two distinct coordinations, peristaltic and synchronous, switches sides periodically every few minutes to ensure that blood circulates equally on the two sides. The same bilaterally paired premotor interneurons and motor neurons produce both coordinations, and in particular only the relative timing of interneurons and motor neurons changes between the two coordinations (Fig. 1*A*; Norris et al., 2007a,b, 2011). It has recently been shown that there are significant side-to-side differences in phasing of activity for each coordination and in underlying synaptic strengths but on a given side, motor output is very similar across repetitions of a coordination (Wenning et al., 2018).

We focus on the coordination of motor neurons (HEs) in segments 8 and 12 that each receive all of their inputs from the same four inhibitory premotor interneurons (HNs) in segments 3, 4, 6, and 7. When one side is in peristaltic coordination, bursting spike patterns of premotor interneurons [HN(3)–HN(7)] show a rear-to-front phase progression (Fig. 1*A*). Bursting phase of premotor interneurons is highly variable across animals and controls the phasing of motor neuron [HE(8) and HE(12)] output spiking patterns (Norris et al., 2007a). In some animals (e.g., in 5/19B), even premotor phase ordering may be different (Fig. 1*B*), but the general trend of rear to front progression is always observed. Variability in premotor input phasing is also reflected by varying motor phases of HE(8) and HE(12) in each animal, but the premotor phase difference (pmΔ*φ*) always sets an upper limit on the forward motor phase progression (mΔ*φ*; see Wright and Calabrese, 2011b). This creates the rear-to-front peristaltic wave constricting heart muscle that is critical for pumping blood and therefore the survival of the leech. Functionality of this feature, despite differing input patterns, depends on the strengths of synapses connecting the interneurons to motor neurons, which also varied across animals.

Previously, Norris et al. (2007a) estimated and reported synaptic strengths using a STA method. Here, we are also reporting within-animal variability of synaptic strengths (Fig. 1*C*) using an improved STA method (see Materials and Methods). Synaptic strengths were normalized per animal to have a meaningful comparison across animals and preparations. When looking across animals, relative strengths were, not only highly variable, but also ordered inconsistently by their magnitudes (Fig. 1*D*). Motor neuron HE(8) had a more even distribution of relative synaptic strengths across all four inputs compared to HE(12) that had small versus large strengths. Across animals, the strongest input to HE(8) alternated between HN(4) and HN(6). Inputs to HE(12) were more consistent across animals, HN(6) and HN(7) being always the strongest; however, their ratios still varied greatly. Other than these rules, synaptic strength patterns seemed to be unique for each animal despite the criticality of their function.

### Functional metrics of the heartbeat circuit such as the motor phase progression are also variable

Functional output metrics of the heartbeat circuit also showed variability across animals in both coordinations (Fig. 2; for a comparison of these distributions to the larger project dataset, see Extended Data Fig. 2-1). These metrics were previously described in detail (Lamb and Calabrese, 2013), and include duty cycle, firing rate, slow wave amplitude, and spike rate. The most important output metric of a functional heartbeat is the peristaltic motor phase progression (mΔ*φ*), which was calculated here as the phase difference from motor neuron HE(8) to HE(12) and exhibited substantial animal-to-animal variability. Variability also existed in the synchronous coordination, but the phase differences were much smaller. Bursting duty cycle and firing rate of the motor neurons were similarly variable across animals. Each animal having different input phase progression, synaptic strengths, and output phase progression, raises the question whether output variability is caused by inputs only, or intrinsic ionic conductances must also differ from animal to animal. Another CPG circuit, the STNS also exhibits similarly stereotypical but variable neuronal output activity in the same neuronal types across individual crustaceans (Bucher et al., 2005), while underlying channel conductances and gene expression patterns vary significantly (Marder and Goaillard, 2006; Schulz et al., 2006). The two to four-fold difference found in maximal conductance magnitudes has been suggested to cause the observed population variability in neuronal activity. Variable underlying conductances in neuronal types was also observed in mammalian Purkinje cells (Swensen and Bean, 2005). We can investigate how changes in inputs and ionic conductances contribute to explaining the outputs in the leech by using a computer model of the heartbeat motor circuit.

### No model instance produced functional output using the measured synaptic weight averages

Previously, Lamb and Calabrese (2013) constructed a conductance-based multicompartmental model of the heart motor neurons (HEs). This is an ensemble model that simulates both sides of the animal because bilateral HE motor neurons are electrically coupled. Premotor input phase and synaptic strength recorded from one side (for both activity coordinations) in experiments were used as inputs to both sides of the model circuit. Synaptic strengths were previously measured by STA experimentally for each HE motor neuron independently (Wright and Calabrese, 2011b). Not knowing how strengths of different HE synapses compare to each other across animals, experimental strengths were interpreted in relative terms. Here, relative synaptic strengths to each HE were scaled by a global scaling parameter (*σ*) to generate absolute strengths to be used in simulations (Fig. 3*A*). The model could then be simulated by playing back inputs from model synaptic currents triggered by recorded HN spike patterns, aiming to recreate HE output phasing that matched recorded animal’s synaptic profiles. This simulation method allows us to explain how the measured properties of the circuit come together to generate motor output and whether it can reproduce observed functional metrics such as burst phase.

10.1523/ENEURO.0417-18.2019.f3-2 Extended Data Figure 3-2 A, Ensemble network model instance showing HE(8) and HE(12) motor neuron firing patterns for peristaltic (magenta traces) and synchronous (blue traces) coordination modes (see explanation of plot in Fig. 3*B*). Points above the traces indicate timing of HN synaptic inputs. Thickness of these points indicate synaptic strength from each input interneuron. In this instance, strengthening synapses for inputs that are earliest (*ρ*_HN3→HE8_ = 4) and latest (*ρ*_HN7→HE12_ = 3) overachieved the peristaltic motor phase progression metric mΔ*φ* = 0.232 *>* 0.105 (5/22B target). ***B***, When repeated for each animal target, theoretical limits of maximal peristaltic motor phase progression followed the magnitude of premotor phase progression. All synapses were zero except *ρ*_HN3→HE8_ = *ρ*_HN7→HE12_ = 4. ***C***, A MOEA was used to search values of the two global *σ* synaptic multipliers to yield errors within target range (blue colored regions) in all metrics (similar to Extended Data Fig. 3-1). Good solution regions for peristaltic motor phase progression (mΔ*φ*, left panel) and the rest of the metrics (right panel) did not overlap, failing to provide any solutions that satisfied low error on all metrics. Worst metric is calculated by finding the MAE across all metrics. Download Figure 3-2, EPS file.

10.1523/ENEURO.0417-18.2019.f3-3 Extended Data Figure 3-3 No MAE solutions within target range (would show as a bright red color) were found after searching across 12,061 combinations of parameter values varied between ranges specified in Table 1. Ion channel maximal conductance (*K*_1_, *K*_2_, *P*, *KCa*, *CaS*, *A*, and *Na*) parameter values were varied between 0 and 50 in somatic, axon, and neurite compartments, electrical coupling (synE) parameter values were varied between 0 and 50, and synapse multiplier (*σ*) parameter values were varied between 0 and 2 values. Ranges marked as 100% indicate the maximal value of that parameter. Download Figure 3-3, EPS file.

Using this method, Lamb and Calabrese (2013) were able to simulate a small motor phase progression target preparation 5/19B (Fig. 1*B*, left-facing triangles) by using measured inputs to the model (Fig. 3*B*). However, the model failed to reproduce larger phase progression targets, such as from 5/22B (Fig. 1*B*, diamonds), using its corresponding inputs (Fig. 3*B*; compare phase progression values in Fig. 2). Theoretically, the model could achieve large motor phase progression when we artificially maximized output phase progression by strengthening earliest (in phase) and latest synaptic inputs to HE(12) and HE(8), respectively (Extended Data Fig. 3-2*A*). Using this method, we confirmed that the output motor phase progression is always limited by, and follows, the input premotor phase progression (Extended Data Fig. 3-2*B*). However, while the model could match larger motor phase progression values, it was unable to keep other functional metrics within target range for any combination of values for the two synaptic scaling parameters (σ) of HE(8) and HE(12), respectively (Extended Data Fig. 3-2*C*).

Furthermore, searching across all maximal conductances (for ionic currents and bilateral electrical coupling between HEs) and HE synaptic scaling parameters of the model failed to find any solutions within all metric target ranges for preparation 5/22B (Extended Data Fig. 3-3). Since intrinsic ionic conductances could not compensate for the model’s shortcomings, we turned back to modifying recorded relative synaptic weights from their average values measured by STA.

### Slightly adjusted synaptic weights result in functional models without changing intrinsic ionic conductances

**Figure 4.**
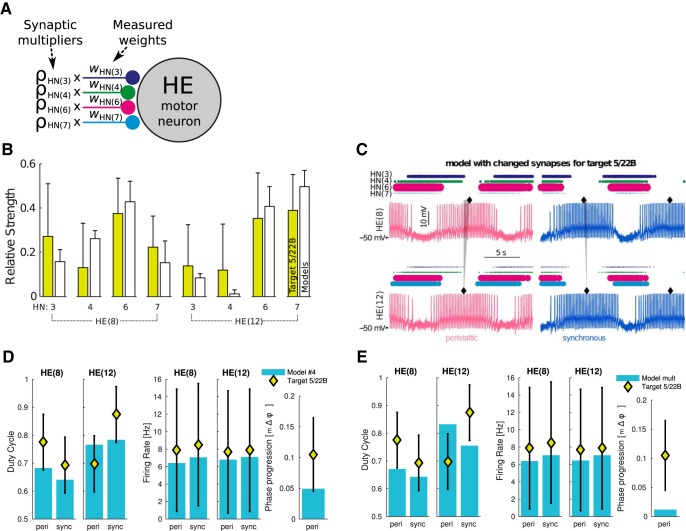
Target 5/22B solution was found by manipulating individual synaptic weights, while keeping the same intrinsic maximal conductance magnitudes of the model instance. ***A***, Individual synaptic multipliers (*ρ*) allow adjusting strengths around recorded averages. ***B***, For input dataset 5/22B, we found 2088 model instances with synaptic weights that are *<*1 within-animal SD of target weights while keeping all other functional metrics also within recorded target ranges. ***C***, Bursting activity of models did not show any abnormalities (example model at rank #4 is shown). ***D***, Metrics of same model compared to target. Error bars show ±1 SD across all the targets. Since synchronous phase progression was small and always within target range, we only show peristaltic progression. ***E***, Same model instance with unmodified target synaptic weights was unable to achieve target values. Figure shows best possible metrics achieved by the model when the global synaptic multipliers were scaled to *σ*_HE8_ = 0.7961 and *σ*_HE12_ = 0.7674.

**Figure 5.**
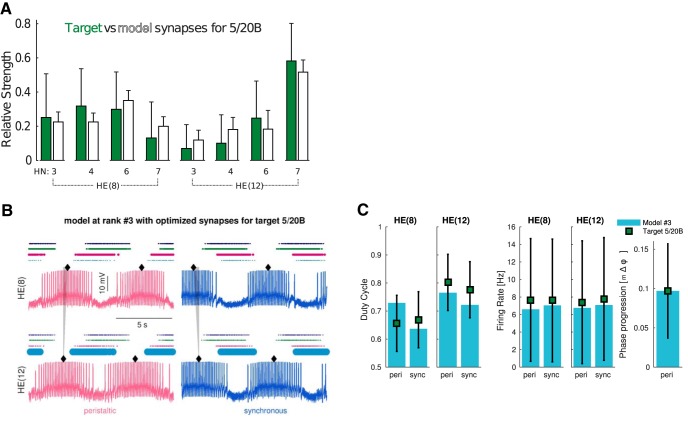
Adjusted synapses for target 5/20B for the model instance that has the same intrinsic maximal conductance magnitudes as in Figure 4. ***A***, Relative synapse strengths (mean ± SD) recorded from preparation 5/20B (green bars) compared to 3717 models (mean ± SD, white bars) found after performing 10,000 MOEA model evaluations. Traces (***B***) and relevant metrics (***C***) of the third highest ranking model.

By preserving the same intrinsic ionic maximal conductance magnitudes in the model instance and allowing synaptic strengths to slightly vary from their measured averages, we were able to find functional models for target 5/22B (Fig. 4) and 5/20B (Fig. 5). To achieve this, we defined synaptic multiplier parameters from each HN input to an HE motor neuron (Fig. 4*A*) and allowed them to vary only within ±1 SD of the recorded synaptic strength averages (Fig. 1*D*). The synaptic strengths found in the top functional models were within ±1 SD of the measured averages for preparation 5/22B (Fig. 4*B*). The simulated models were able to produce the target phase progression of the individual animal with no other electrophysiological anomalies (Fig. 4*C*). The measured functional metrics were all within target ranges as well (Fig. 4*D*). If we had used the original measured synaptic strength averages, the metrics would be outside of this range for the synchronous duty cycle of HE(12) and much lower than the target of the peristaltic motor phase progression (Fig. 4*E*). To show another example, we were able to find synaptic strengths (Fig. 5*A*) and functional output metrics (Fig. 5*C*) within ±1 SD of the measured targets for preparation 5/20B that produced proper bursting patterns (Fig. 5*B*). These two examples gave us confidence for searching nearby synaptic strengths to those measured in the rest of the animals.

### Adjusting synaptic strengths resulted in functional models for all six preparations

**Figure 6.**
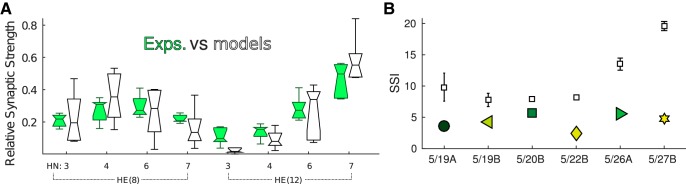
Summary of model synaptic strengths found for all targets. Bursting activity was regular across all preparations (Extended Data Fig. 6-1). ***A***, Boxplots compare distributions of synaptic strengths of recorded (Exps.) versus best performing model (Models) across all six animals. Extended Data Figure 6-2 shows synaptic strengths found for individual animals. ***B***, Synaptic strength index (SSI) compared across targets and mean ± SE of top 10 models (black boxes).

We repeated the procedure for the remaining four preparations and obtained functional models by finding new synaptic strengths within ±1 SD of measured averages (Fig. 6). The best motor phase progression errors obtained were *<*0.6 of its target range across all preparations and largest error was associated with different metrics in each one (Table 4). Bursting activity was also regular across all preparations (Extended Data Fig. 6-1). When compared across six animals, we found no significant difference between relative synaptic strength distributions of measured averages versus topmost models (Kruskal–Wallis, *p >* 0.05; Fig. 6*A*). Furthermore, the model synaptic weights followed the same relative pattern (same distribution) across eight inputs as in living HE neurons (for synaptic strengths found for individual animals, see Extended Data Fig. 6-2). To explain the significance of the synaptic weight pattern, we used the synaptic strength index (SSI) defined by Wright and Calabrese (2011b). The SSI metric calculates the sum of preferences of HN(4) to HE(8) and of HN(7) to HE(12) in a single animal by using

$$\text{SSI}\;=\;\frac{w_{HN4\to HE8}}{w_{HN4\to HE12}}+\frac{w_{HN7\to HE12}}{w_{HN7\to HE8}}$$

where *w* are relative synaptic strengths. When we applied this equation to both measured and model synaptic strengths for all preparations, we found that model SSI values increased for preparations with larger phase progression, while measured SSI values remained almost flat (Fig. 6*B*; see Discussion). Despite this discrepancy and having reproduced the observed peristaltic phase progression values in the model without sacrificing any other functional metric, we turned to the question of whether intrinsic ionic conductances also contributes to individual variability across animals. Surprisingly, all the results we presented so far (except Extended Data Fig. 3-3) were obtained with the same combination of ionic conductances in the model. This suggests that synaptic strengths dominate the phasing of the output and ionic conductances need not vary across animals. Next, we investigated which combinations of ionic conductances resulted in functional models while keeping the newly found synaptic strengths constant.

**Table 4.** Synaptic multiplier search for all six preparations

Preparation	Target mΔ*φ*	Best mΔ*φ*	Error mΔ*φ*	MAE caused by
5/19A	0.07	0.0591	−0.18	+0.58 in peri spike height for HE(12)
5/19B	0.08	0.0592	−0.36	+0.77 in sync duty cycle for HE(12)
5/20B	0.09	0.0788	−0.30	−0.66 in peri slow wave height for HE(12)
5/22B	0.07	0.0962	−0.15	−0.95 in peri slow wave height for HE(12)
5/26A	0.14	0.1619	+0.28	−0.76 in peri slow wave height for HE(8)
5/27B	0.17	0.1414	−0.59	+0.61 in sync slow wave height for HE(12)

Best mΔ*φ* value and its error are from the best out of top five lowest MAE (MAE in any metric) models. All top models were within target range error for all metrics. MAE column is for the top model.

10.1523/ENEURO.0417-18.2019.f6-1 Extended Data Figure 6-1 HE(8) and HE(12) model membrane voltage traces of example ensemble model instances for synaptic configurations shown in Figure 6. Traces are displayed the same way as in Figure 3*B*. Proper bursting patterns were observed for each of the six preparations (panels ***A–F***). For each preparation, ensemble models only with best matching peristaltic phase progression (mΔ*φ*) are shown. Download Figure 6-1, EPS file.

10.1523/ENEURO.0417-18.2019.f6-2 Extended Data Figure 6-2 Details of the relative synaptic weight summary statistics given in Figure 6*A*. Average and standard error of relative synaptic weights from the top 10 ranking models (empty squares with error bars) compared to those measured (filled symbols) for all six animal targets. Symbols map to animal targets same as throughout this article (Fig. 1). Consistent with the summary graph, in all examples model synaptic strengths followed trends from measured values. Arrows intended only for labeling the symbols. Download Figure 6-2, EPS file.

### Intrinsic conductances vary smoothly around synaptic solutions found

So far, we have tested all animal targets with models that employed the same one configuration of intrinsic conductances. To test the impact of intrinsic conductance deviation from this configuration in the heart motor neuron output across animals, we varied intrinsic conductances without allowing changes in synaptic strengths from the newly found values. As expected, we found ensembles of model solutions for each of the animals (Table 5). For each animal, we evaluated at least 5000 model instances in randomized MOEA searches. Model instances within target range (“good” models) were just a small percentage of the total number of instances evaluated, varying between 1% and 13%. This reinforced the understanding that intrinsic conductances are important for producing functional network activity. However, we wanted to assess the scale of this importance and whether intrinsic conductances must vary across animals. Therefore, we asked whether there are other fixed sets of intrinsic conductances that can produce model instances that function across animals. We selected animal 5/22B, which was the most difficult phase target to achieve and which had the lowest ratio of good models. We tested each of its 122 good model instances against other animal targets by setting synaptic strengths to the previously found values for that animal and keeping them constant. At least some of these instances produced network activity patterns within target ranges for all animals (Table 6, best MAE varied between 0.51 and 0.70). And >50% of these instances worked in all other animals except target 5/27B (only 28% of 5/22B good model instances were also good for target 5/27B). Having found several intrinsic conductance sets that work across animals supported our view of their secondary role in individual variation of network activity.

**Table 5.** Results of model searches in intrinsic conductance parameters

Target	# Good	*r_g_* (%)	*R* ^2^	*p*
5/19A	656	13.13	0.78	3.01 × 10^−235^
5/19B	347	6.94	0.53	2.99 × 10^−62^
5/20B	140	2.81	0.84	2.91 × 10^−61^
5/22B*	122	1.19	0.86	8.40 × 10^−54^
5/26A	199	3.99	0.81	2.20 × 10^−75^
5/27B	461	9.23	0.50	2.79 × 10^−76^

To avoid overfitting, searches were divided into separate batches, each starting from random initial conditions. For each animal, five batches of searches were executed with 1000 model instance evaluations in each. *r_g_* is the ratio of good (functional) models to all evaluated models. *5/22B was searched with five batches of 2000 evaluations. Linear regression was applied to check for correlation between K_2_ and P channel conductances, for which statistics are presented (*R* ^2^ and *p* values).

**Table 6.** Functional models (*n* = 122) from target animal 5/22B were simulated with synaptic strengths and input patterns of other animals and checked for whether they were within their target ranges

Target	MAE range	# Good	Ratio (%)
5/19A	0.51–1.16	104	85
5/19B	0.70–1.76	62	50
5/20B	0.64–1.70	74	60
5/26A	0.60–1.34	101	82
5/27B	0.68–1.99	35	28

Range of metric MAE shows closeness of activity to target range (<1 accepted as good), “# Good” shows number of functional models, and ratio is the percentage of good models out of 122.

**Figure 7.**
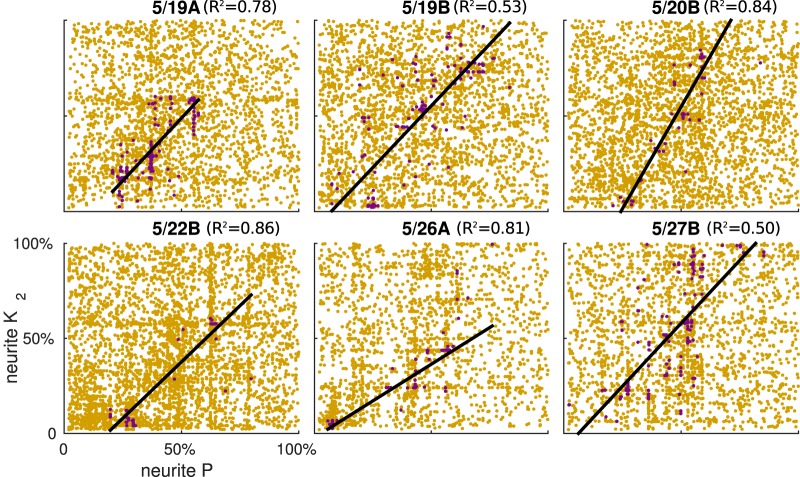
Linear regression fits (black lines) were similar across all preparations for functional models (burgundy dots) among a background of 5000 evaluated maximal intrinsic ionic conductance combinations (yellow dots) between neurite K_2_ and P channel conductances. Regression resulted in *R* ^2^ *>* 0.5 for all preparations, shown in each panel’s title. All *p* values were <0.01 (Table 5).

Next, we looked at correlation properties across intrinsic conductances in the good sets of model instances that we had found for each animal independently (Table 5). Hudson and Prinz (2010) had suggested that ionic conductance ratios may represent neuronal type and must remain constant across individuals of a population. Here, we provide support for this hypothesis by finding a similar positive correlation pattern across six preparations (Fig. 7) for one of the known ionic conductance pairs (delayed-rectifier K_2_ and persistent sodium P current in the neurite compartments) that was previously shown to be correlated (Lamb and Calabrese, 2013). We looked for intrinsic conductance correlations in the same data that we presented above (Tables 5, 6). We have found other intrinsic conductance correlations in our dataset, but focused on one conductance pair to perform comparisons across animals.

## Discussion

One of the principal questions in neuroscience is how reliable functional neuronal output can be produced while underlying neuronal and synaptic properties are variable across members of a species (Hamood and Marder, 2014). CPG neuronal networks have provided excellent opportunities to study this question (Marder and Calabrese, 1996). Studies across animals focused on properties such as constancy of phase (Hooper, 1997; Bucher et al., 2005; Goaillard et al., 2009; Hamood et al., 2015), period regulation (Kushinsky et al., 2019), and morphologic variability (Otopalik et al., 2017) in the pyloric and cardiac motor patterns of the STG of crustaceans. Similar studies have focused on swimming motor pattern in the lamprey (Grillner, 1974) and on the crayfish swimmeret system (Smarandache et al., 2009). In the leech heartbeat system, the functional output is the phase difference between motor neuron discharge (bursts) in different segments, which control blood circulation through heart tubes. Within functional limits, the phase of the activity patterns of the premotor interneurons and motor neurons, and the synaptic strengths between them, are variable across individuals (Norris et al., 2011), the effects of which are not well understood. Computer models give us the ability to combine disparate experimental measurements to simulate circuit function, which can be used to explore these interactions (Wright and Calabrese, 2011b). We set the parameters of a heart motor neuron circuit model, controlled by the heartbeat CPG, to data collected from a set of six individuals. Our results showed that synaptic strengths play a critical role in producing highly variable animal-specific motor output burst phasing. This is not surprising as the inhibitory synapses from premotor CPG interneurons are responsible for shaping the phasing of the output (Wright and Calabrese, 2011a,b). This is consistent with other studies that look at the interplay between synapses and intrinsic conductances in living neurons across animals (Williams et al., 2013; Anwar et al., 2015; Lane et al., 2016) and across instances of neuronal circuit models (Prinz et al., 2004; Doloc-Mihu and Calabrese, 2011; Williams et al., 2013; Lane et al., 2016). In particular, Lane et al. (2016) showed that, in the crustacean cardiac ganglion, removing one intrinsic conductance can be compensated by changes in another intrinsic conductance and in strength of electrical coupling. When we tested the opposite relation in our model, modification of synaptic strengths could not be compensated by changes in intrinsic membrane conductances.

In our neuronal network model, the values of synaptic strengths measured (as averages in STA) previously did not produce functional output, but we were able to find nearby values within the error of the STA that did. Once functional synaptic weights were found, ionic conductances could be varied to generate ensemble model sets. Outside of these solution sets, models produce non-functional output even with the functional synaptic strengths. Thus, while synaptic strengths predominate in determining output phasing, membrane ionic conductances play an important role in tuning and translating that synaptic input into functional coordinated neuronal output. These results potentially apply to many other motor networks where CPGs exert strong synaptic control over motor neurons.

### Why did using measured synaptic strengths fail to generate functional circuit models?

Since both activity patterns and synaptic strengths were recorded from the same individuals, it was surprising to find that network model outputs did not match measured features when we used the measured STAs of the strengths. There are several possible explanations for this failure.

Using average values from disparate measurements is known to cause some errors if values come from concave spatial distributions (Goldman et al., 2001; Golowasch et al., 2002). The STA is a well-established method that produces a single averaged value (in this case, synaptic strength) and therefore is not subject to this problem (Ito, 2015). However, if individual EPSCs averaged in the STA come from a bimodal distribution, the resulting averages may be unphysiological. We had already improved the accuracy and increased the signal-to-noise ratio of the STA method employed here (see Materials and Methods), although there could be remaining sources of variability in terms of synaptic plasticity or baseline shifts that may be causing pernicious errors in the measurements. A small error in parameter values can unbalance a whole model particularly in a situation like that here where it is the relative strengths of inputs that is key; a misestimate in one input can throw off the relative strengths.

Another explanation could be a missing feature in the model, although this is unlikely based on previous scrutiny. The heart motor neuron circuit model is constructed with a high level of biological detail based on physiologic properties measured specifically from the leech. It contains ten ionic currents characterized from voltage clamp recordings; multiple electrical compartments to represent important anatomic distinctions and spatial separation for the axon, soma, and neurites; chemical synapses that feature short-term plasticity, electrical coupling between the heart motor neurons, and conduction delays for intersegmental spikes arriving from premotor interneurons ( García et al., 2008; Wright and Calabrese, 2011b; Lamb and Calabrese, 2013).

### The same configuration of model intrinsic conductances can create output patterns of multiple animals when synaptic strengths are near measured (STA) values

The same set of intrinsic ionic conductances used in an instance of the HE motor neuron ensemble model was able to reproduce the individually varying output phasing measured from each of the six individual leeches. Usually, activity in identified neuron types are stereotypical across individuals, but with individual variation. This is in contrast to observed 2- to 4-fold variability of intrinsic ionic conductances found in neurons of one type across animals (Bucher et al., 2005; Goaillard et al., 2009). In both of these studies, animals were collected from a natural population that presumably contained significant biological variability. Thus, it is not surprising to observe variability also in neuronal properties, and it can be attributed to environmental, genetic, or developmental factors. However, from a functional network output perspective, is it necessary for these individuals to have different intrinsic ionic conductance magnitudes? Could one transplant the same neuron into another individual and expect to match their individual functional output? Since we cannot answer this question easily experimentally, the closest we can get is through computer simulation.

In addition to showing that one set of intrinsic conductances can produce functional network activity across animals, we also showed that one can find multiple such intrinsic conductance configurations. To achieve this, we evaluated thousands of models in the intrinsic conductance space. Only a small percentage of these models produced functional models (Table 5). This confirmed that intrinsic conductances are also important in generating functional network output. We further showed that some of these intrinsic conductance configurations can produce the observed output in multiple animals. Not all of the configurations we selected from one animal produced the observed (target) activity in all animals, possibly because they were selected randomly and not by specific criteria. These results strongly suggest that one can find a large number of model intrinsic conductance configurations that can produce the individually unique functional activity of multiple animals.

Achieving functional output despite observed variability of neuronal intrinsic ionic conductances in wild-caught animals, for example in neurons of the crab STNS, have been attributed to co-regulation rules for maximal conductances based on electrophysiological recordings (Golowasch et al., 1999a,b) and mRNA counts (Schulz et al., 2006, 2007). Co-regulated intrinsic conductances found in neuron types thus often compensate for each other and produce stereotypical activity patterns (Hamood et al., 2015; Lane et al., 2016). Therefore, although intrinsic conductances are variable, output produced is similar, which supports our finding that a single type of neural activity produced by the same set of intrinsic model parameters could function in multiple animals as long as the relative synaptic strengths are appropriate to the individual. Within error limits, the relative synaptic strengths could also vary in a large number of model solutions. By definition, relative synaptic strengths imply that, across neuronal models, absolute values are linearly related. Therefore, the synaptic strengths that we found using the model are also correlated. Having already confirmed experimentally some of these predictions (Norris et al., 2011), our model results can be taken to further analyses.

In a computational modeling study of the crustacean STG, Hudson and Prinz (2010) hypothesized that characteristic neuronal types must have similar co-regulation rules. However, this conclusion was reached by constructing ensemble neuronal models that match population targets rather than data from individual animals. Ensemble neuronal modeling creates a large set of neuronal models that fit a certain output criterion and mine it for biological questions (Prinz, 2010; Doloc-Mihu and Calabrese, 2011; Günay, 2015). Here, we provide support for the hypothesis of Hudson and Prinz (2010) by constructing ensemble model databases of the heartbeat motor neuron circuit model across animals. Once we established the new individually appropriate synaptic strengths, we kept them constant and varied intrinsic ionic conductances to find sets able to match each animal target. In the resulting ensemble datasets, we showed that some intrinsic ionic conductances exhibit similar co-regulation rules across animals, supporting the earlier prediction and suggesting a required motor neuron type for functional motor output.

In summary, we find that synaptic strengths are critical in creating the proper output phasing in a rhythmic motor circuit. Rhythmic circuits are responsible for many types of motor activity such as walking, chewing, breeding, and breathing across many species and are critical for life (Marder and Calabrese, 1996). The type of CPG motor circuit that we studied here is similar to circuits in other species and our results may extend beyond the leech. Understanding parameters and limits of animal-to-animal variability would help explain and compensate for artifacts when consistent measurements are required in higher-order animals such as rats (Falcone et al., 2018). Our conclusion is that animals in the wild seem to cope with neuronal and network variability by achieving custom solutions to reach functional output. This is consistent with other studies that show that observed individual variability may not have a significant functional impact (Otopalik et al., 2017). As a result, each individual may have unique combination of synaptic parameters that enable signature stereotypical behavior they express.

## Synthesis

Reviewing Editor: Muriel Thoby-Brisson, CNRS UMR 5287 Université Bordeaux Decisions are customarily a result of the Reviewing Editor and the peer reviewers coming together and discussing their recommendations until a consensus is reached. When revisions are invited, a fact-based synthesis statement explaining their decision and outlining what is needed to prepare a revision will be listed below. The following reviewer(s) agreed to reveal their identity: Jorge Golowasch.

Dear Authors,

After an intense discussion with the two reviewers, who actually got interest in your study but a completely different opinion on the overall interpretation, we ended up with a consensus on the major comments they have on the manuscript. These comments are summarized in the following text:

1- The hypothesis tested in this study should be more clearly stated. Indeed the title suggests that intrinsic conductances do not play a role, which is not true; in the intro the sentence “how do the network and intrinsic parameters interact to create functional output across animals remain vague and does not fully fit with the experiments developed in the paper. In fact the sentence in Significance Statement: “Here, we propose that, for a rhythmic neuronal motor circuit, the uniqueness derives mainly from differences in synaptic strength rather than from differences in intrinsic membrane conductances.” would be the clearer statement for the hypothesis tested here. So the first comment would be to formulate the hypothesis of the work in a much more precise manner.

2- Related to this unclear hypothesis, as the paper is written the reader might be frustrated going along the text because he could expect a completely different set of results examining more specifically the interaction between intrinsic conductances and synaptic strength to produce a functional output. Because this does not seem to be the goal of the study, despite being an interesting complementary question, it should be made clear that interaction between cellular and network properties in producing the motor output will not be addressed in the paper. Another possibility would be to also add this issue in the revised version, but this might require additional experiments and/or analysis.

3- Figure 7 is very confusing and should be more thoroughly described and interpreted.

4- The study is based on a low number of preparations (n=6). The authors should argue why only 6 and how they have been chosen.

To conclude, the reviewers request a more thorough description of what figure 7 shows, a clearer hypothesis, and a clearer conclusion (in the abstract and elsewhere) about what the relative roles of synaptic strength and intrinsic currents are. Thus the authors should consider to re-write the paper to be more objective about the hypothesis, the interpretation, and the conclusions.

Below are the detailed comments:

Major comments :

1.) The study lacks a clear hypothesis. The title suggests that intrinsic conductances “need not vary across animals.” And yet they clearly do. The study proceeds solely from the point of view of generating a single canonical output for each individual, and thus is far too limited to make a determination as to the role of variability in conductances. Therefore, it is not clear whether the authors claim that variability in intrinsic conductances are artifactual? Or a “developmental” signature that is irrelevant to steady state output? The field of variable network parameters has largely moved on to focus on robustness of network output, and the role that variability and modulation of conductances (both intrinsic and synaptic) may play in these regards. Without a defined hypothesis, it is not clear what advance this study makes over others that have examined how a single canonical output might be generated. A much more direct hypothesis -evidence-conclusion structure to this work may reveal more meaningful insight.

2. The strongest potential in this study is the suggestion by the authors that they attempt to ask “how do the network and intrinsic parameters interact to create functional output across animals?” Yet this experiment is not performed. Holding synaptic strength constant and varying intrinsic conductances, and vice versa, does not examine interaction. Co-varying synaptic and intrinsic conductance to determine how they interact - particularly under different “environmental” conditions - would provide meaningful insight. If studies were performed to truly look at interactions among membrane and synaptic conductance, it would greatly enhance the contribution of the manuscript.

3. The authors base their study and conclusions about population level variability on six preparations (N=6), which is a modest number given the current power of computational approaches. Further, it is not clear how these six were chosen, how representative they are of the population (what distribution of output characteristics and underlying properties they represent), and by what criteria their inclusion in the study was determined. It is difficult for the reader to determine how meaningful these six individuals are relative to the broad scale with which the interpretations of these experiments are put forth.

4. Figure 7 is not super clear nor super thorough: While I agree that synaptic currents will dominate phasing, the linear regression results presented in figure 7 seem to suggest that ionic currents are very much relevant. Given the similarities in the location of the models for the six target outputs, it seems that deviations from a narrow band of possibilities would lead to substantial variation. Furthermore, the authors do not report how many models in which conductances were varied were rejected for not producing the appropriate output. If a substantial number were rejected, then this is evidence the conductances contribute more strongly to phase than implied in this study. It is not clear from the manuscript whether any of the models which did not meet requirements for 5/19A, for instance, would satisfy the targets for another. If this is not the case it would bolster the author's argument.

Related to the previous comment, if it is the case that variation in network output does not depend on nor is affected by variable underlying neurons (or rather is less sensitive to changes in these parameters relative to synaptic ones), then we would expect that any of the model neurons shown in figure 7 should produce the target activity of any of the other five networks given their pre-determined synaptic strengths. In other words, are neurons across the six model networks interchangeable? Is this the case for a high percentage of these models or do they fail to produce any of the target activities? Simulations in which neurons can wholesale be “swapped” across networks and still generate appropriate output would be supportive of the authors' case.

Also, why don't the authors show how other currents behave, and whether that the correlations and partial correlations they showed previously still hold. However, the way I interpreted Fig. 7 was that modifying those 2 currents by more than one SD away from average would cause something to fail in their model without necessarily producing a complete crash. Is this correct?

Minor comments :

-The authors use the term “ionic conductances” to refer to intrinsic ionic conductances and distinguish them from synaptic conductances. Perhaps the word “intrinsic” would be useful since synaptic currents are strictly also ionic.

-p. 3, line 2 in 1st paragraph: This line should read “to escape from ...”

-p. 13, line 6 in section titled “Why did using ...” I do not understand the sentence “The STA is a well-established method that is not known to have this weakness” in relation to the sentence before. The STA is a method that generates a single average value of something (here, synaptic strength). The errors generated in averaging values of concave distributions stems from the fact that these distributions involve at least 2 parameters or variables. Can you explain how these two sentences relate?

-p. 13, second subtitle is missing “the” before “model”

- In Figure 1, line 5 there are two numbers are absent.

- In multiple sections, they authors allude to “development” and “homeostasis.” This is not well developed or integrated enough into the hypothesis - nor are any citations provided - to warrant such broad strokes. “Our conclusion is that animals in the wild seem to cope with the variability they face during development and environmental effects by using rules to achieve custom solutions to reach functional output.” This is not set up in a meaningful way, and yet is a very important question. This should be developed and supported directly by the data, or a different framework employed.

- The authors state in the first paragraph of the Results that ““Within-animal variance of HE(12) synaptic strengths was larger for some preparations, which could be attributed to the largeness of their averages.” Why then would you not use a normalized measure of variance, such as coefficient of variation?
